# A Clash of Old and New Scientific Concepts in Toxicity, with Important Implications for Public Health

**DOI:** 10.1289/ehp.0900887

**Published:** 2009-07-30

**Authors:** John Peterson Myers, R. Thomas Zoeller, Frederick S. vom Saal

**Affiliations:** 1 Environmental Health Sciences, Charlottesville, Virginia, USA; 2 Biology Department, University of Massachusetts, Amherst, Massachusetts, USA; 3 Division of Biological Sciences, University of Missouri, Columbia, Missouri, USA

**Keywords:** biphasic, bisphenol A, dose–response curve, inverted U, low dose, nonmonotonic, regulatory toxicology

## Abstract

**Background:**

A core assumption of current toxicologic procedures used to establish health standards for chemical exposures is that testing the safety of chemicals at high doses can be used to predict the effects of low-dose exposures, such as those common in the general population. This assumption is based on the precept that “the dose makes the poison”: higher doses will cause greater effects.

**Objectives:**

We challenge the validity of assuming that high-dose testing can be used to predict low-dose effects for contaminants that behave like hormones. We review data from endocrinology and toxicology that falsify this assumption and summarize current mechanistic understanding of how low doses can lead to effects unpredictable from high-dose experiments.

**Discussion:**

Falsification of this assumption raises profound issues for regulatory toxicology. Many exposure standards are based on this assumption. Rejecting the assumption will require that these standards be reevaluated and that procedures employed to set health standards be changed. The consequences of these changes may be significant for public health because of the range of health conditions now plausibly linked to exposure to endocrine-disrupting contaminants.

**Conclusions:**

We recommend that procedures to establish acceptable exposure levels for endocrine-disrupting compounds incorporate the inability for high-dose tests to predict low-dose results. Setting acceptable levels of exposure must include testing for health consequences at prevalent levels of human exposure, not extrapolations from the effects observed in high-dose experiments. Scientists trained in endocrinology must be engaged systematically in standard setting for endocrine-disrupting compounds.

The very public debate about potential harmful consequences of exposure to the plastic monomer bisphenol A (BPA) is a leading high-profile battleground in a scientific revolution currently under way in toxicology (Layton 2008; Myers et al. 2009). But much more is under contention than the health risks of one chemical. Data emerging from studies of endocrine-disrupting chemicals (EDCs), such as BPA, that mimic or in numerous ways interfere with hormone action, challenge the central assumption that has guided toxicology for centuries, including today’s regulatory apparatus for assessing chemical safety. In so doing, they challenge the methods and the adequacy of chemical exposure safety standards.

## Using High-Dose Testing to Predict Low-Dose Effects

The core assumption of regulatory toxicology is that experiments using high doses will reveal potential effects of low doses. This is derived from 16th-century dogma but is still typically applied today by federal regulators (White et al. 2009), although it conflicts directly with well-established principles in endocrinology regarding hormone action. The acceptance of this assumption has profound implications for the assessment of risk to human health posed by EDCs.

The approach of using very high-dose testing to predict consequences of much lower doses that are typically within the range of widespread human exposure emerges from a 16th-century observation by Paracelsus that toxicologists paraphrase as “the dose makes the poison” (Gallo 1996). Paracelsus’ logic holds if and only if a chemical’s effects follow a monotonic dose–response curve, in which more of the chemical leads to a greater effect. Monotonicity and nonmonotonicity refer to changes in the slope of the curve describing dose and response. Monotonic curves may be linear or nonlinear, but the slope never reverses from positive to negative or vice versa. The slope of a nonmonotonic curve changes sign, from positive to negative or vice versa. Biologically relevant nonmonotonic curves include “U-shaped” or “inverted-U–shaped” dose–response relationships. When toxicologists began to focus on potential health effects of EDCs, endocrinologists raised questions about the appropriateness of assuming monotonicity as a basis for chemical risk assessments, because nonmonotonicity is a general characteristic of endogenous hormones, hormonally active drugs, and environmental chemicals with hormonal activity.

Indeed, Paracelsus’ assumption is directly contradicted by decades of research in endocrinology and clinical medicine showing that hormonally active compounds have dose–response curves in which low doses can cause effects opposite to those at high doses. This issue is so central to hormone action that it is a critical component of determining the dose required for hormonally active drugs. Two well-known examples are Lupron [used to treat reproductive disorders in women and men (Garner 1994)] and tamoxifen [used to treat breast cancer (Mortimer et al. 2001)], in which low doses stimulate whereas high doses inhibit disease. Specifically, for both of these drugs, a phenomenon known as low-dose “flare” occurs, during which there is stimulation of the response that the drug inhibits when the blood level of the drug reaches the high clinically effective dose range (e.g., for Lupron, testosterone secretion in men with prostate cancer; and for tamoxifen, proliferation of mammary tissue in women with breast cancer).

## Nonmonotonic Dose–Response Curves

Nonmonotonic dose–response curves result from multiple mechanisms. Hormones and hormone-mimicking chemicals act through receptors in target cells. Very low doses can stimulate the production of more receptors (receptor up-regulation), resulting in an increase in responses, whereas higher doses (within the typical toxicologic range of chemical testing) can inhibit receptors (receptor down-regulation), resulting in a decrease in responses (Welshons et al. 2003). The consequence for gene activity, which is regulated by hormone-mimicking chemicals binding to receptors that amplify very small exposures into very large responses, is that very low doses of these chemicals (in the case of a positively regulated gene) can up-regulate gene expression, whereas at higher doses the same chemicals down-regulate gene expression (Coser et al. 2003; Medlock et al. 1991; Vandenberg et al. 2007).

If only one response is being measured, a nonmonotonic dose–response curve is a common finding for EDCs. An additional complication, however, is that when multiple outcomes are examined, qualitatively different outcomes are commonly observed at low and high doses of EDCs. One basis for this is that the suite of genes whose expression is regulated by low doses of endogenous hormones and chemicals that mimic these hormones can be completely different from the genes affected by high doses (Coser et al. 2003). As the dose increases, hormones and hormone-mimicking chemicals can bind to receptors for other hormones, referred to as receptor cross-talk. For example, at high doses, endogenous and man-made environmental estrogens begin to interact with androgen and thyroid hormone receptors, producing entirely different effects from those seen at low doses, when only significant binding to estrogen receptors occurs (Welshons et al. 2003). Furthermore, myriad hormonal feedback mechanisms among the brain, pituitary gland, and hormone-producing organs (e.g., thyroid gland, adrenal glands, ovaries, testes) contribute to the presence of nonmonotonic dose–response curves and qualitatively different responses at low and high doses of EDCs. The consequence is that high doses and low doses differ not just in quantitative effects but also in qualitative impact, especially when responses of whole organisms are considered.

Another consideration is that the effects of EDCs classified as “xenoestrogens” are not identical. As research has progressed into identifying the molecular mechanisms mediating responses, a consensus has emerged that this class of EDCs should be categorized as selective estrogen receptor modulators, to highlight the fact that each can result in a unique array of responses. However, conducting studies that involve comparing activities of different xenoestrogens (or other chemicals that act via similar mechanisms) requires understanding the importance of the doses being used (Shioda et al. 2006).

EDCs may also act by mechanisms that do not require direct mediation by classical hormone receptors. Nonspecific (non–receptor-mediated) toxicity can occur at high but not low doses. EDCs also exert actions upon synthesis or function of enzymes that may be responsible for the synthesis or degradation of hormones and on coregulatory proteins that interact with receptors and, in the case of neurologic actions, affect neuro-transmitters and their receptors (Gore 2007). For example, low doses of atrazine activate aromatase gene activity in zebrafish embryos; this activity can alter sex determination via a rapid signaling system (Suzawa and Ingraham 2008). This concept is important because each of these mechanisms may have a unique dose–response relationship for a particular EDC, adding to the complexity of the overall shape of the dose–response curve for each response.

Of great importance, above the dose at which a hormonally active chemical saturates (occupies virtually all) receptors, any change in response that occurs cannot be caused by a receptor-mediated mechanism, which requires a change in receptor occupancy. Receptors for steroid hormones are ligand-activated transcription factors that require a change in ligand binding to affect the rate of gene transcription. Thus, high-dose experiments cannot be used to predict low-dose results mediated by EDCs binding to hormone receptors and altering receptor-mediated responses at low doses. The current paradigm in regulatory toxicology of only testing a few very high doses of chemicals within a relatively narrow dose range (with the highest dose being the maximum tolerated dose) thus does not serve to predict the hazards posed by low-level exposure to numerous EDCs found in most people in biomonitoring studies conducted in the United States and elsewhere (Calafat et al. 2008).

**Figure 1:**
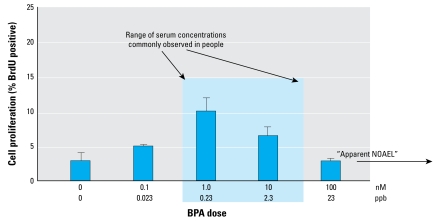
BPA induces cell proliferation in androgen-independent LNCaP prostate cancer cells. LNCaP cells were propagated for 72 hr in 5% charcoal-/dextran-treated fetal bovine serum supplemented with 0.1% ethanol vehicle and increasing BPA concentrations (0.1–100 nM). Cells were then labeled with bromode-oxyuridine (BrdU), and BrdU incorporation was detected via indirect immunofluorescence. Data shown are mean ± SD of three independent experiments in which at least 250 cells/experiment were analyzed. The shaded region indicates typical concentrations found in humans (Vandenbergh et al. 2007). The response to 100 nM BPA did not differ from control. A standard toxicity test, working down the dose–response curve from high doses, would have shown no difference between controls and exposed animals at a dose at that level or above and would have used it to identify the “apparent no observed adverse effect level (NOAEL),” indicated by the arrow. Testing at lower doses would not have been conducted, and the stimulatory effect of BPA at 1 nM and 10 nM would never have been observed. Figure modified from Wetherill et al. (2002).

Nonmonotonic dose–response curves have been reported for adverse effects with a number of EDCs (Myers and Hessler 2007), including the polycarbonate plastic monomer BPA (Figure 1) used in some baby bottles, water bottles, and food can linings (Wetherill et al. 2002); di(2-ethylhexyl) phthalate (DEHP), used in medical devices and other products made with polyvinyl chloride plastic (Takano et al. 2006); and the pesticides dieldrin, endosulfan, and hexachlorobenzene (Narita et al. 2007). For example, exposure to DEHP at a concentration 1,000-fold less than the current safety standard, which is based on high-dose liver toxicity, exacerbated allergic reactions (Takano et al. 2006). Similarly, exposure to extremely low (picomolar, parts per trillion) levels of several persistent organic pollutants increased allergic responses (Narita et al. 2007). None of these effects was predicted by studies that examined only high doses of these chemicals.

In an experiment explicitly designed to test the adequacy of high-dose testing of DEHP in rats, Andrade et al. (2006) found that a high dose increased estrogen-synthesizing (aromatase) enzyme activity in the brains of neonatal male rats; a dose 100-fold lower appeared to be the “no effect dose,” which is used to estimate the dose deemed safe for human exposure (the aromatase enzyme is involved in determining sex differences in brain function). Only because the scientists broke with tradition and also tested lower doses did they find significant down-regulation of aromatase at a dose 37 times lower than the putative no effect dose, an effect opposite to and unpredicted from results of testing only very high doses.

Other experiments have documented nonmonotonicity in rat pituitary and cerebellar cortex cells exposed to picomolar through micromolar levels of BPA (Wozniak et al. 2005; Zsarnovszky et al. 2005). Acting through a relatively recently discovered non-classical estrogen response system, very low picomolar concentrations of BPA increased calcium influx and activation of enzyme cascades that dramatically amplify a very low-dose signal into a large cellular response. The dose–response curve followed a nonmonotonic inverted-U shape, with the strongest response at picomolar to low nanomolar levels. The bioactive concentrations of BPA in these experiments were below the range found ubiquitously in human blood and urine. Other end points that follow a nonmonotonic pattern for BPA are human prostate cancer cell proliferation (Figure 1) (Wetherill et al. 2002), promotion of human seminoma cell proliferation (Bouskine et al. 2009), and production of the insulin-response–regulating hormone adiponectin by human adipocytes (Hugo et al. 2008). These specific responses to BPA occurred within the range of human exposure to BPA based on biomonitoring studies (Calafat et al. 2008; Richter et al. 2007; Schonfelder et al. 2002) but were not observed at much higher doses.

Research over the past 20 years has identified multiple EDCs that mimic or disrupt hormone function at low doses in ways that are not predicted by high-dose studies. Biomonitoring studies have established that many of these contaminants are widespread in people. Yet classical regulatory toxicology ignores nonmonotonicity despite the fact that, similar to hormones, EDCs would be expected to display nonmonotonic dose–response patterns for many responses. This disconnect with current science pervades virtually all regulatory agencies responsible for chemical safety around the world, and it means that many regulatory decisions are highly likely to have underestimated risks.

## Health Implications

If the health implications of these decisions were inconsequential, the clash between regulatory toxicology and endocrinology would appropriately remain buried in academia. But the range of health conditions now plausibly linked to EDCs—including, but not limited to, prostate cancer (Chamie et al. 2008), breast cancer (Soto et al. 2008), attention deficit hyperactivity disorder (Ishido et al 2004), infertility and male and female reproductive disorders (Hauser and Sokol 2008; Swan 2008), miscarriage, and most recently, hyperallergic diseases, asthma (Bornehag et al. 2004), obesity (Hugo et al. 2008), and heart disease and type 2 diabetes (Lang et al. 2008; vom Saal and Myers 2008)—makes it imperative that the clash between endocrinology and regulatory toxicology be resolved in ways that reflect modern scientific understanding.

These chronic diseases are major contributors to the steadily increasing human disease burden and to the escalating cost of health care throughout the world. Extensive, careful, and replicable animal research suggests that numerous common man-made chemicals to which people are exposed every day, but that have not been adequately studied for health effects in humans, may be significant contributors to these adverse health trends. Because the endocrine system is highly conserved between animals used as models in biomedical research and humans, the default assumption should be that nonmonotonic dose–responses of EDCs observed in laboratory animals and *in vitro*, including with human cells and tissues, are applicable to human health (Hugo et al. 2008; Wetherill et al. 2007). Modernizing relevant health standards by incorporating endocrinologic principles could help reduce a significant portion of the human disease burden, but this will require regulatory decision makers to fundamentally change the paradigm commonly used to assess the risk to human health posed by chemicals.

## Specific Recommendations and Conclusion

We recommend the following:

- Animal testing protocols used to establish regulatory safety standards must include experiments that examine effects of chemicals over a wide dose range that at their low end overlap with typical human exposures, particularly those experienced by vulnerable populations based on biomonitoring data, or modeling if actual data do not exist.
- Current scientific knowledge obtained through studies on the endocrine system and its disruption by exogenous chemicals should be applied systematically when regulatory standards on EDCs are to be established. For the best interest of public safety, cooperation of chemical manufacturers in reevaluating safety of their products under the new criteria is critical. Their acceptance of the endocrinology-derived concept that high-dose experiments are insufficient to establish safety standards for EDCs is essential. Continued denial of the reality that nonmonotonic dose–response curves are predicted to occur for EDCs is no longer tenable (Bird 2005; vom Saal 2005).

The soaring health care crisis unfolding in countries around the world demands that the regulatory apparatus of governments move into the 21st century. Blind obedience to 16th-century dogma will not solve the problem. Unless and until regulatory agencies incorporate modern endocrinologic principles into their risk assessment paradigms, they will continue to provide false assurances of “safety” and fail to recognize the actual health risks posed by chronic low-level exposure to an increasing number of chemicals found in commonly used products.

## References

[b1-ehp-117-1652] Andrade AJ, Grande SW, Talsness CE, Grote K, Chahoud I (2006). A dose-response study following in utero and lactational exposure to di-(2-ethylhexyl)-phthalate (DEHP): nonmonotonic dose-response and low dose effects on rat brain aromatase activity. Toxicol.

[b2-ehp-117-1652] Bird J (2005). Hyperbole or commonsense. Chem Ind.

[b3-ehp-117-1652] Bornehag CG, Sundell J, Weschler CJ, Sigsgaard T, Lundgren B, Hasselgren M (2004). The association between asthma and allergic symptoms in children and phthalates in house dust: a nested case–control study. Environ Health Perspect.

[b4-ehp-117-1652] Bouskine A, Nebout M, Brucker-Davis F, Benahmed M, Fenichel P (2009). Low doses of bisphenol A promote human seminoma cell proliferation by activating PKA and PKG via a membrane G-protein-coupled estrogen receptor. Environ Health Perspect.

[b5-ehp-117-1652] Calafat AM, Ye X, Wong LY, Reidy JA, Needham LL (2008). Exposure of the U.S. population to bisphenol A and 4-tertiary-octylphenol: 2003–2004. Environ Health Perspect.

[b6-ehp-117-1652] Chamie K, DeVere White RW, Lee D, Ok JH, Ellison LM (2008). Agent Orange exposure, Vietnam War veterans, and the risk of prostate cancer. Cancer.

[b7-ehp-117-1652] Coser KR, Chesnes J, Hur J, Ray S, Isselbacher KJ, Shioda T (2003). Global analysis of ligand sensitivity of estrogen inducible and suppressible genes in MCF7/BUS breast cancer cells by DNA microarray. Proc Natl Acad Sci USA.

[b8-ehp-117-1652] Gallo MA, Klaassen CD (1996). History and scope of toxicology. Casarett and Doull’s Toxicology: The Basic Science of Poisons.

[b9-ehp-117-1652] Garner C (1994). Uses of GnRH agonists. J Obstet Gynecol Neonatal Nurs.

[b10-ehp-117-1652] Gore AC (2007). Introduction to endocrine-disrupting chemicals. Endocrine-Disrupting Chemicals: From Basic Research to Clinical Practice.

[b11-ehp-117-1652] Hauser R, Sokol R (2008). Science linking environmental contaminant exposures with fertility and reproductive health impacts in the adult male. Fertil Steril.

[b12-ehp-117-1652] Hugo ER, Borcherding DC, Gersin KS, Loftus J, Ben-Jonathan N (2008). Prolactin release by adipose explants, primary adipocytes, and LS14 adipocytes. J Clin Endocrinol Metab.

[b13-ehp-117-1652] Ishido M, Masuo Y, Kunimoto M, Oka S, Morita M (2004). Bisphenol A causes hyperactivity in the rat concomitantly with impairment of tyrosine hydroxylase immunoreactivity. J Neurosci Res.

[b14-ehp-117-1652] Lang IA, Galloway TS, Scarlett A, Henley WE, Depledge M, Wallace RB (2008). Association of urinary bisphenol A concentration with medical disorders and laboratory abnormalities in adults. JAMA.

[b15-ehp-117-1652] Layton L (2008). Studies on chemical in plastics questioned. Washington Post.

[b16-ehp-117-1652] Medlock KL, Lyttle CR, Kelepouris N, Newman ED, Sheehan DM (1991). Estradiol down-regulation of the rat uterine estrogen receptor. Proc Soc Exp Biol Med.

[b17-ehp-117-1652] Mortimer JE, Dehdashti F, Siegel BA, Trinkaus K, Katzenellenbogen JA, Welch MJ (2001). Metabolic flare: indicator of hormone responsiveness in advanced breast cancer. J Clin Oncol.

[b18-ehp-117-1652] Myers JP, Hessler W (2007). Does ‘the dose make the poison?’. Environ Health News.

[b19-ehp-117-1652] Myers JP, vom Saal FS, Akingbemi BT, Arizono K, Belcher S, Colborn T (2009). Why public health agencies cannot depend on Good Laboratory Practices as a criterion for selecting data: the case of bisphenol A. Environ Health Perspect.

[b20-ehp-117-1652] Narita S, Goldblum RM, Watson CS, Brooks EG, Estes DM, Curran EM (2007). Environmental estrogens induce mast cell degranulation and enhance IgE-mediated release of allergic mediators. Environ Health Perspect.

[b21-ehp-117-1652] Richter CA, Birnbaum LS, Farabollini F, Newbold RR, Rubin BS, Talsness CE (2007). In vivo effects of bisphenol A in laboratory rodent studies. Reprod Toxicol.

[b22-ehp-117-1652] Schonfelder G, Wittfoht W, Hopp H, Talsness CE, Paul M, Chahoud I (2002). Parent bisphenol A accumulation in human maternal–fetal–placental unit. Environ Health Perspect.

[b23-ehp-117-1652] Shioda T, Chesnes J, Coser KR, Zou L, Hur J, Dean KL (2006). Importance of dosage standardization for interpreting transcriptomal signature profiles: evidence from studies of xenoestrogens. Proc Natl Acad Sci USA.

[b24-ehp-117-1652] Soto AM, Vandenberg LN, Maffini MV, Sonnenschein C (2008). Does breast cancer start in the womb?. Basic Clin Pharmacol Toxicol.

[b25-ehp-117-1652] Suzawa M, Ingraham HA (2008). The herbicide atrazine activates endocrine gene networks via non-steroidal NR5A nuclear receptors in fish and mammalian cells. PLoS ONE.

[b26-ehp-117-1652] Swan SH (2008). Environmental phthalate exposure in relation to reproductive outcomes and other health endpoints in humans. Environ Res.

[b27-ehp-117-1652] Takano H, Yanagisawa R, Inoue K, Ichinose T, Sadakane K, Yoshikawa T (2006). Di-(2-ethylhexyl) phthalate enhances atopic dermatitis-like skin lesions in mice. Environ Health Perspect.

[b28-ehp-117-1652] Vandenberg LN, Hauser R, Marcus M, Olea N, Welshons WV (2007). Human exposure to bisphenol A (BPA). Reprod Toxicol.

[b29-ehp-117-1652] vom Saal FS (2005). Low-dose BPA: confirmed by extensive literature. Chem Ind.

[b30-ehp-117-1652] vom Saal FS, Myers JP (2008). Bisphenol A and risk of metabolic disorders. JAMA.

[b31-ehp-117-1652] Welshons WV, Thayer KA, Judy BM, Taylor JA, Curran EM, vom Saal FS (2003). Large effects from small exposures. I. Mechanisms for endocrine-disrupting chemicals with estrogenic activity. Environ Health Perspect.

[b32-ehp-117-1652] Wetherill YB, Akingbemi BT, Kanno J, McLachlan JA, Nadal A, Sonnenschein C (2007). In vitro molecular mechanisms of bisphenol A action. Reprod Toxicol.

[b33-ehp-117-1652] Wetherill YB, Petra CE, Monk KR, Puga A, Knudsen KE (2002). The xenoestrogen bisphenol A induces inappropriate androgen receptor activation and mitogenesis in prostate adenocarcinoma cells. Mol Cancer Ther.

[b34-ehp-117-1652] White RH, Cote I, Zeise L, Fox M, Dominici F, Burke TA (2009). State-of-the-science workshop report: issues and approaches in low-dose-response extrapolation for environmental health risk assessment. Environ Health Perspect.

[b35-ehp-117-1652] Wozniak AL, Bulayeva NN, Watson CS (2005). Xenoestrogens at picomolar to nanomolar concentrations trigger membrane estrogen receptor-α-mediated Ca^2+^ fluxes and prolactin release in GH3/B6 pituitary tumor cells. Environ Health Perspect.

[b36-ehp-117-1652] Zsarnovszky A, Le HH, Wang HS, Belcher SM (2005). Ontogeny of rapid estrogen-mediated extracellular signal-regulated kinase signaling in the rat cerebellar cortex: potent non-genomic agonist and endocrine disrupting activity of the xenoestrogen bisphenol A. Endocrinology.

